# A robust and resilience machine learning for forecasting agri-food production

**DOI:** 10.1038/s41598-022-26449-8

**Published:** 2022-12-16

**Authors:** Reza Lotfi, Amin Gholamrezaei, Marta Kadłubek, Mohamad Afshar, Sadia Samar Ali, Kiana Kheiri

**Affiliations:** 1 Department of Industrial Engineering, Yazd University, Yazd, Iran; 2 Behineh Gostar Sanaye Arman, Tehran, Iran; 3 Department of Industrial Engineering, Iran University of Science and Technology, Tehran, Iran; 4 Faculty of Management, Department of Logistics, Czestochowa University of Technology, Czestochowa, Poland; 5 Department of Industrial Engineering, Central Tehran Branch, Islamic Azad University, Tehran, Iran; 6 Department of Industrial Engineering, Faculty of Engineering, King Abdulaziz University, Jeddah, Saudi Arabia; 7 Department of Computer Science, Utah State University, Utah, USA

**Keywords:** Applied mathematics, Computational science

## Abstract

This research proposes a new framework for agri-food capacity production by considering resiliency and robustness and paying attention to disruption and risk for the first time. It is applied robust stochastic optimization by adding robustness to the constraint's objective function and resiliency situation. This research minimizes the mean absolute deviation and coefficient of standard deviation errors by linear function in the agri-food capacity production. This study suggests agri-food managers and decision-makers use this mathematical method to forecast and improve production management. The results of this research lead to better decision-making and are compared with other sine functions. The main model's Robust and Resiliency Mean Absolute Deviation (RRMAD) value is 1.28% lower than other sine-type functions. The conservativity coefficient, confidence level, weight factor, resiliency coefficient, and probability of the scenario vary. The main model's RRMAD value is 1.28% lower than other sine-type functions. Growing the weight factor will result in an increase in RRMAD and a smooth decline in *R-squared*. Additionally, as the resilience coefficient rises, the RRMAD function increases while the *R-squared* declines. By altering the probability of the scenario, the RRMAD function drops, and the *R-squared* goes up.

## Introduction

Forecasting the amount of production is a critical factor for business life^1–3^. In addition, predicting the production volume can manage production capacity and tackle disruptions like COVID-19, natural disasters, and man-made carefully^4,5^.

Regarding the complex situation in Iran (including COVID-19, natural disasters, and natural man-made), this issue causes the supply of the material to have a problem for production. As a result, the production number decreases, and many unsatisfied demands exist in the supply chain^6,7^. Eventually, researchers need to predict production by considering the complex situation to help all supply chains to decrease costs and move toward producing without disruption and estimate the actual situation^8,9^.

**Figure 1:**
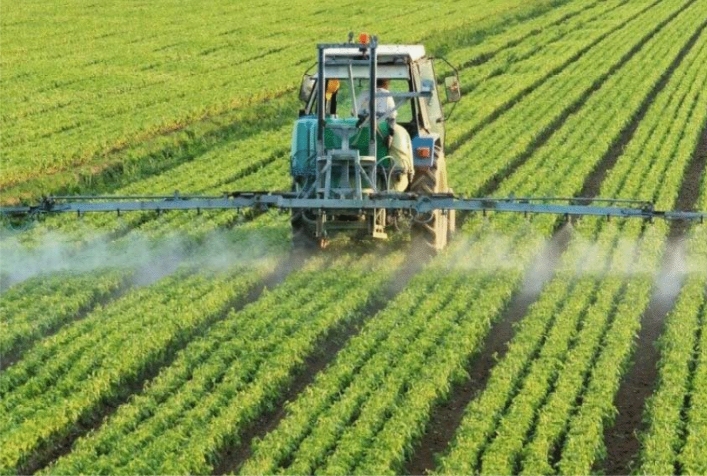
Agri-food production^10^.

The literature review applies tools and methods to forecast data^11,12^. Machine learning (ML) is a cutting-edge methodology rapidly gaining popularity. This study plan to use ML tools to predict the total agri-food production in Iran (cf. Fig. 1). These tools help supply chain managers and government policymakers forecast production capacity and demand^13^. In this investigation want to use a new robust regression approach to tackle uncertainty and generate a suitable model to predict the volume of production in the country until policymakers manage the hard situation.

Accordingly, the main contributions of this research are given as follows:

- A new Robust and Resilience ML (RRML) approach,
- Projecting production by using a new robust regression approach,
- Considering hard and complex conditions for predicting resilience against disruption.

The remaining sections are organized as follows. In Section "Survey on related work", various models for complex events are analyzed. Section "Problem description" explains the problem and develops the proposed regression-based robust optimization model. The actual case study and computational results are presented in Section "Results and discussion". Managerial insights and practical implications of the study are discussed in Section "Managerial insights and practical implications", and finally, Section "Conclusions and outlook" concludes the main achievements and limitations and provides a helpful outlook for the research.

## Survey on related work

Numerous methods are used in the literature review to forecast the volume of production and demand. Every tool has advantages and disadvantages. Before finding the best solution, researchers should carefully choose the best model to predict. After reviewing and surveying the ML approach, there is no paper to contribute to the RRML. As a result, this study is likely the first to examine RRML. However, using ML for forecasting has been the subject of extensive research. As a result, the following are looked to model types:

### Simple model

This section examines the simple model for forecasting using ML. Simple models only utilize one method to predict and show trends. Kantasa-Ard, Nouiri^14^ utilized ML for demand forecasting on the physical internet. They embedded this model in agricultural products in Thailand. They used a Long Short-Term Memory (LSTM) with a hybrid Genetic Algorithm (GA) and Scatter Search (SS) to tune the parameters of the LSTM. Pereira and Cerqueira^15^ applied ML regression methods for forecasting hotel demand to manage revenue. They employed 22 methods for determining short-term demand forecasting with a 14-day lead time. They proposed Arbitrating ML as a meta-learning approach by combining the dynamic ensemble method. They found that using the ML method decreased the mean square error by 54%.

Kohli, Godwin^16^ applied a linear and KNN regression for forecasting sales. Using this method, they can predict sales and plan for resilience against disruption and fluctuation. Ali et al.^17^ measure green supply chain management's environmental and sustainable impact by using manufacturing organization survey data. The authors developed a sustainability framework using machine learning-based CHAID analysis to reduce environmental damage and improve the organization's business performance. However, they used the PLS-SEM package with 380 data responses from various manufacturers. Item Response Theory post hoc analysis is used to confirm the scope and effectiveness of the measurement model after additional robustness of the proposed model is validated using various ML (machine learning) techniques^18^.

Papacharalampous and Langousis^19^ presented Quantile Regression Algorithms (QRA) for water demand forecasting. They used probabilistic thinking to cope with uncertainty and compared the method with quantile regression, linear boosting, generalized random forest, gradient boosting machine, and quantile regression neural network algorithms. In addition, They applied this model to urban water flow.

BV and Dakshayini^20^ applied machine learning tools to the project market, used Multiple Linear Regression (MLR) and an ANN model, and attempted to forecast demand in agriculture. They found the proposed helpful model reliable and quiet for planning and producing agri-food. Baryannis, Dani^21^ surveyed supply chain risks and presented the ML approach to predict supply chain risks. They establish to define a trade-off between performance and interpretability. They used Data-Driven Artificial Intelligence (AI) techniques to estimate supply chain risks. Lotfi, Kheiri^22^ developed a novel approach based on robust regression to predict the number of patients with COVID-19 in Iran. They utilized robust convex optimization and Mean Absolute Deviation (MAD) to forecasting patients of COVID-19. They compared the model with the well-known model and showed that the new model's performance was better than the previous model.

### Hybrid model

This section describes the hybrid model for forecasting in the ML approach. The hybrid model applies several methods to predict and show trends. The results show that the hybrid model is usually more efficient than the simple model. Carbonneau, Laframboise^23^ suggested using ML techniques for supply chain demand forecasting. They proposed Neural Networks (NN), Recurrent Neural Networks (RNN), and Support Vector Machines (SVM) for predicting demand in the supply chain. Fradinata, Kesuma^24^ used a Support Vector Regression (SVR) and Adaptive Neuro-Fuzzy (ANFIS) to measure the Bullwhip Effect (BE) in the supply chain. They used the model described above to reduce the impact of the bullwhip effect and discovered that ANFIS performed better than SVR in terms of Mean Square Error (MSE) and Bias Error (BE).

Al-Musaylh, Deo^25^ reviewed multiple-horizon forecasting for electricity demand. They implemented a novel strategy for Queensland, Australia that involved combining a two-phase Particle Swarm Optimized SVR (PSOSVR) hybrid model with enhanced empirical mode decomposition and adaptive noise.

For demand forecasting at the retail stage for a few vegetables, Priyadarshi, Panigrahi^26^ used a Box–Jenkins-based auto-regressive integrated moving average model along with ML-based algorithms like Long Short-Term Memory (LSTM) networks, SVR, random forest regression, Gradient Boosting Regression (GBR), and extreme GBR (XGBoost/XGBR).

Kilimci, Akyuz^27^ proposed a deep learning strategy and decision integration approach for demand forecasting in the supply chain. They proposed an innovative method based on Deep Learning (DL) techniques, the SVR algorithm, and time series and employed this methodology to forecast demand.

Phyo and Jeenanunta^28^ demonstrated daily load forecasting using a combination of Classification and Regression Tree (CART) and Deep Belief Network (DBN). They applied this model for load data in the Electricity Generating Authority of Thailand (EGAT). Yucesan, Pekel^29^ proposed regression, time series, and ML-based methods for forecasting daily natural gas consumption. They applied the Seasonal Autoregressive Integrated Moving Average with Exogenous Regressors (SARIMAX) and Artificial Neural Networks (ANN). A novel ML approach for demand forecasting and supply chain performance was developed by Feizabadi^30^. In this research, ARIMAX and NN are developed and applied by steel manufacturers.

### Research gap

Based on the application of the ML approach, the relevant works are organized and reviewed in Table 1. As is evident, our goal is to design RRML, which has not yet been developed. The most pertinent research in the literature is categorized in Table 1, along with comparisons of the methodology, case study(s) or scenarios, and goal.

**Table 1:** Related works.

Reference	Type	Methodology	Predicting	Case study
^23^	Hybrid	NN, RNN, SVM	Demand	Canada
^24^	Hybrid	SVR, ANFIS	Demand	Tin milk industry
^25^	Hybrid	PSOSVR	Electricity demand	Queensland, Australia
^21^	Simple	Data-driven AI	Supply chain risks	Numerical Example (NE)
^27^	Hybrid	DL, SVR, time series	Demand forecasting	SOK Market in Turkey
^26^	Hybrid	LSTM, SVR, XGBoost/XGBR	Demand forecasting	Vegetables
^28^	Hybrid	CART, DBN	Load data	EGAT
^14^	Hybrid	LSTM, GA, SS	Demand agricultural products	Thailand
^15^	Simple	Regression	Hotel revenue	South of Europe
^29^	Hybrid	SARIMAX, ANN	Natural gas consumption	NE
^16^	Simple	KNN regression	Sale	NE
^22^	Simple	Robust regression	COVID-19	Iran
^19^	Simple	QRA	Urban water flow	NE
^30^	Hybrid	ARIMAX, ANN	Demand	Steel manufacturer
^20^	Hybrid	MLR, ANN	Demand	Agri-food
**This research**	**Simple**	**RRML**	**Forecast production**	**Agri-food production, Iran**

Significant values are in bold.

Given the research gap in Table 1, the main novelty of this study is RRML for predicting production in the future and considering supply problems. In other words, it is necessary to design a model to predict production under its complicated uncertainty that can be efficiently utilized in future decision-making processes.

**Figure 2:**
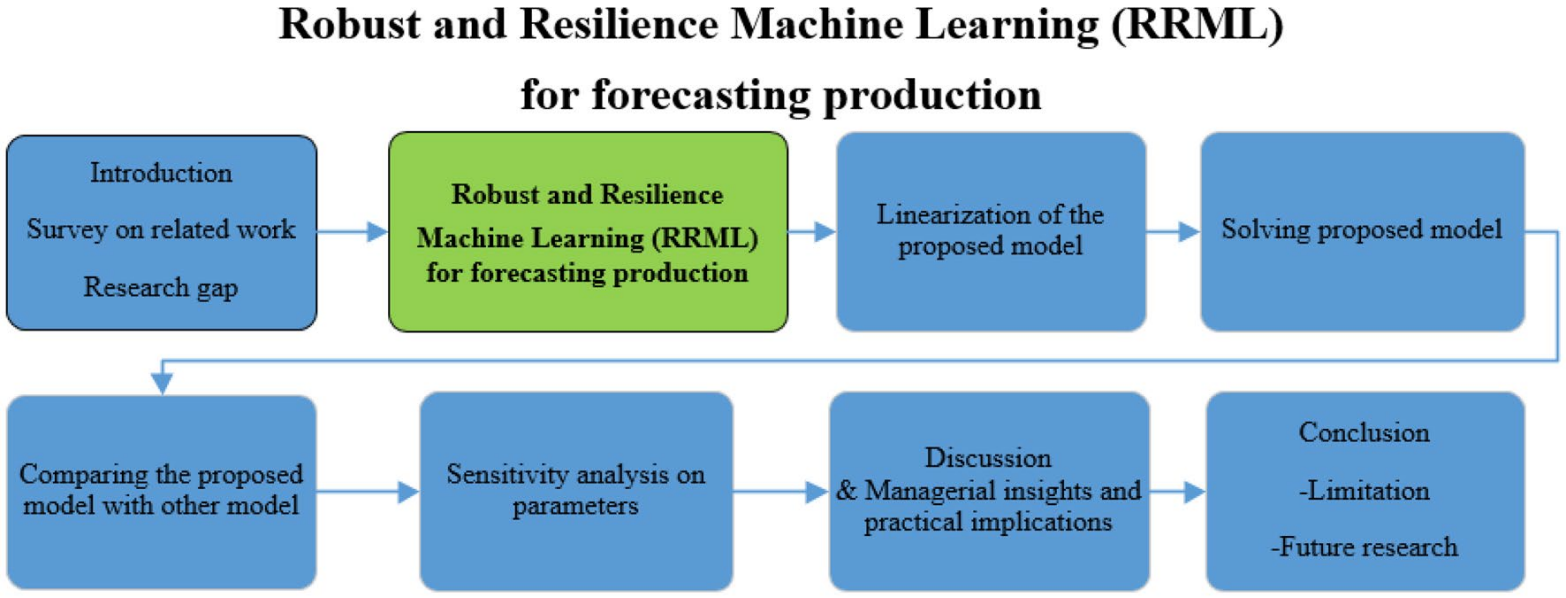
Research methodology.

Figure 2 is a flow chart that is drawn to describe the research methodology and the steps of the suggested method. The significance of this research can be summed up as a novel approach for production projection called Robust and Resilience ML (RRML), which considers hard and complex conditions for disruption resilience by using a robust regression approach.

The contribution of this research is as follows:

- A new Robust and Resilience ML (RRML) approach,
- Projecting production by a new robust regression approach,
- Considering hard and complex conditions for resilience against disruption.

## Problem description

**Figure 3:**
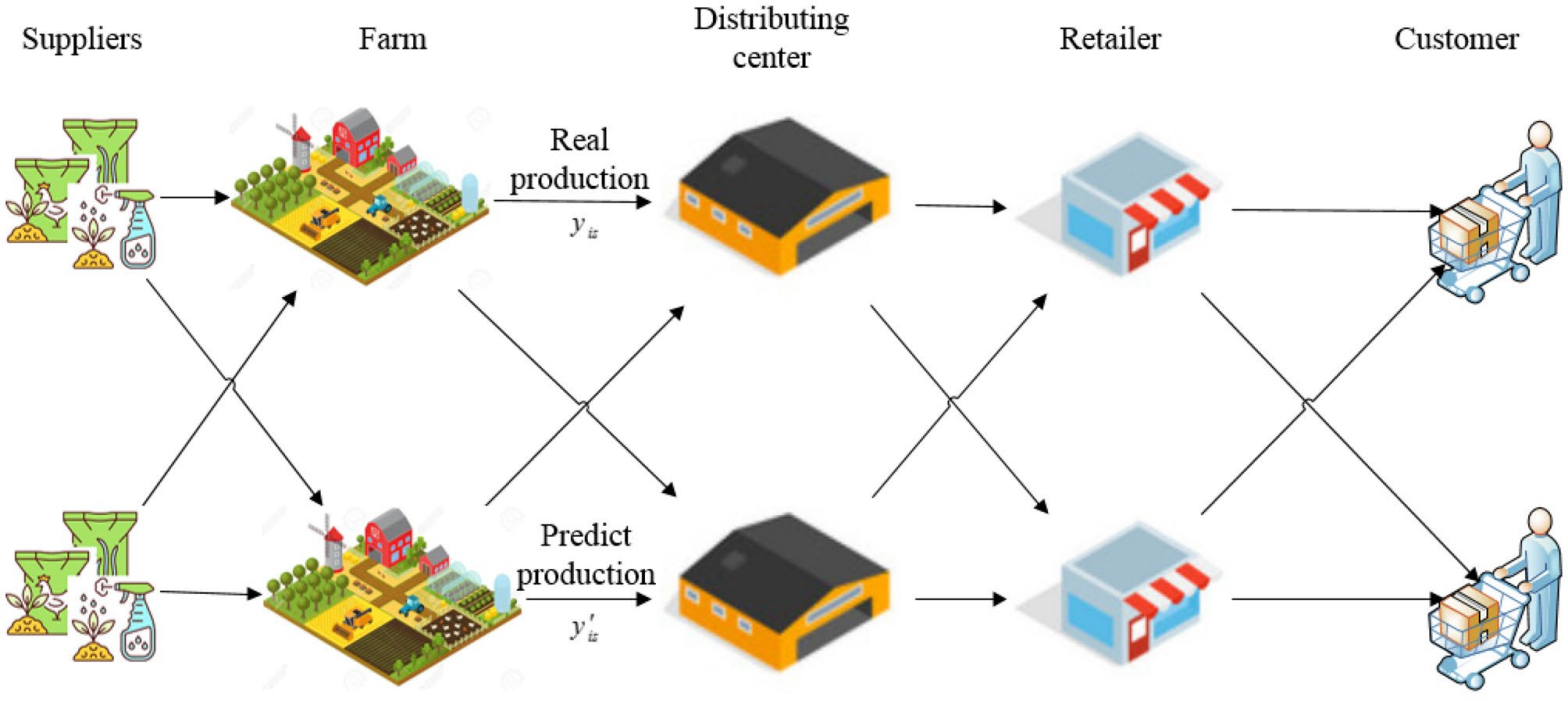
RRML for forecasting production.

This study attempt to forecast the quantity of agri-food production based on the years. The aim of research plan and forecast agri-food production until those in charge of decision-making in the agri-food sector can enable good decisions and define policy. Despite data uncertainty, the forecast between year ($x_{i}$) and production ($y_{is}$) for various scenarios is estimated (cf. Fig. 3). Although there is uncertainty in data, it is estimated to forecast production ($y^{\prime}_{is}$) under scenarios. This section introduces the proposed model for long-term forecasting. Therefore, relative years and number of production are considered for projecting the production. This research uses robust stochastic optimization to predict the volume of production in Agri-food production. Eventually, it is suggested RRML based on this scope:

- Robust approach: Robust stochastic programming for regression-based,
- Resiliency: considering the resiliency coefficient depend on the scenario as a resiliency approach against disruption.

### Mathematical model

Consequently, it is necessary to make the following assumption:

Assumption:

- There is a stochastic nature (uncertainty) in the data.
- There is no dependence between the data.

In the following, the indices, parameters, and variables to develop the proposed model are defined first:

Notations:

**Indices**
$$i$$
Index of years; $i \in I = \{ 1,2,...,\overline{i}\}$
$$k$$
Index of regression degree; $k \in \{ 1,...,\overline{k}\}$
$$s$$
Index of scenario $s \in S = \{ 1,2,...,\overline{s}\}$

**Parameters**
$$x_{i}$$
Year counter (Year *i*),
$$y_{is}$$
Number of production in year $i$ under scenario $s$
$$p_{s}$$
Probability of scenario $s$
$$\rho_{s}$$
Resiliency coefficient under scenario $s$
$$\beta$$
Conservatitvity coefficient
$$w_{i}$$
Weight factor of error $i$
$$\alpha$$
Confidence level
$$\lambda$$
Weight factor of old and new data
$$\omega$$
Cycle period

**Variables**
$$y^{\prime}_{is}$$
Number of forecasted production in year $i$ under scenario $s$
$$\Gamma_{is}$$
Absolute deviation between real and forecasted production in year $i$ under scenario $s$
$$\overline{\Gamma }_{i}$$
Mean deviation between real and forecasted production in year $i$
$$\overline{\sigma }_{i}$$
Standard deviation between real and forecasted production in year $i$
$$f_{s} (x_{i} )$$
Proposed function forecasted production in year $i$ under scenario $s$
$$RRMAD$$
Robust and Resilience Mean Absolute Deviation (RRMAD)

Model 1 RRML for production forecasting:

1 $${\text{min}}\,\,RRMAD = \beta \sum\limits_{i} {w_{i} \overline{\Gamma }_{i} } + (1 - \beta )\sqrt { - 2\ln (\alpha )} \sum\limits_{i} {w_{i} \overline{\sigma }_{i} } ,$$

subject to

Distance and risk constraint:

2 $$\Gamma_{is} = \left| {y^{\prime}_{is} - y_{is} } \right|\quad \forall i,s$$

3 $$\overline{\Gamma }_{i} = \sum\limits_{s} {p_{s} } \Gamma_{is} ,\quad \forall i,s$$

4 $$\overline{\sigma }_{i} = \sum\limits_{s} {p_{s} } \left| {\Gamma_{is} - \overline{\Gamma }_{i} } \right|,\quad \forall i$$

Resiliency constraint:

5 $$y^{\prime}_{is} = \rho_{s} f_{s} (x_{i} ),\quad \forall i,s$$

6 $$f_{s} (x_{i} ) = \sum\limits_{k} {a_{k} x_{i}^{k - 1} } \quad \forall i,s$$

Weight constraint:

7 $$\sum\limits_{i} {w_{i} } = 1,$$

8 $$w_{i} = \lambda \frac{i}{\left| I \right|(\left| I \right| + 1)/2} + (1 - \lambda )\frac{\left| I \right| - (i - 1)}{{\left| I \right|(\left| I \right| + 1)/2}},\quad \forall i$$

The objective function (1) tries to minimize the RRMAD. The RRMAD includes the mean absolute deviation and coefficient of standard deviation between real and forecasted production in all years. Constraint (2) represents the deviation between real and forecasted production in year $i$ under scenario $s.$ Constraint (3) states the mean forecasted production in year $i.$ Constraint (4) states the standard deviation of forecasted production in year $i.$ Constraint (5) shows the amount of forecasted production in year $i$ under scenario $s.$ Constraint (6) presents linear regression function that must be fitted. Therefore, polynomial regression is suggested. Constraint (7) considers the summation of weight factors must be one. Constraint (8) offers a new form of weight factor that is tuned by old and new data.

### Linearization of the proposed model

Given the absolute function in Model 1, it should be linearized using the following equations. Therefore, two new positive variables are employed to linearize the absolute function.

If $k = \left| {\Omega_{s} } \right|,$ therefore, the following replacements can be considered in the model:$k = \alpha_{s} + \beta_{s} ,\,\,\,\,\Omega_{s} = \alpha_{s} - \beta_{s} ,\,\,\,\alpha_{s} ,\beta_{s} \ge 0,\,\,\forall s.$

Linearization of RRML:

$${\text{min}}\,\,RRMAD = \beta \sum\limits_{i} {w_{i} \overline{\Gamma }_{i} } + (1 - \beta )\sqrt { - 2\ln (\alpha )} \sum\limits_{i} {w_{i} \overline{\sigma }_{i} } ,$$

subject to

9 $$\Gamma_{is} = va_{is} + vb_{is} ,\quad \forall i,s$$

10 $$y^{\prime}_{is} - y_{is} = va_{is} - vb_{is} ,$$

11 $$\overline{\sigma }_{i} = \sum\limits_{s} {p_{s} } (va^{\prime}_{is} + vb^{\prime}_{is} ,),\quad \forall i$$

12 $$\Gamma_{is} - \overline{\Gamma }_{i} = va^{\prime}_{is} - vb^{\prime}_{is} ,\quad \forall i,s$$

13 $$va_{is} ,vb_{is} ,va^{\prime}_{is} ,vb^{\prime}_{is} \ge 0,\quad \forall i,s$$

Constraints (3), (5)–(8)

### Complexity of the problem

The complexity of linearization of RRML includes numbers of binary, positive and free variables and constraints as indicated in Eqs. (14) to (17). As can be seen, one of the essential factors for constraints, positive and free variables, is scenario sets. Positive, free variables and constraints are linear in the relation between scenarios:

14 $${\text{Binary}}\,{\text{variables number}} = 0,$$

15 $${\text{Positive}}\,{\text{variables number}} = 4\left| I \right|\left| S \right|,$$

16 $${\text{Free}}\,{\text{variables number}} = 2\left| I \right| + 3\left| I \right|\left| S \right| + \left| K \right| + 1,$$

17 $${\text{Constraints number}} = 5\left| I \right|\left| S \right| + 2\left| I \right| + 1.$$

This model has no binary variable and is completely LP. As a result, the large scale of this problem is solved in polynomial time. Consequently, increasing scenarios make to increase time polynomially.

### Correlation coefficient of the proposed model

After estimating the parameters of polynomial regression, it is needed to measure dependency and quality of response. Then, the scenario-based correlation coefficient ($R^{2}$) is employed to measure the quality of response (RRMAD). Finally, the scenario-based correlation coefficient is calculated according to Eqs. (18), (19):

18 $$RS_{s}^{2} = 1 - \frac{{\sum\limits_{i} {(y_{is} - y^{\prime}_{is} )^{2} } }}{{\sum\limits_{i} {(y_{is} - \frac{1}{\left| I \right|}\sum\limits_{i} {y_{is} } )} \,^{2} }},$$

19 $$R^{2} = \sum\limits_{s} {p_{s} RS_{s}^{2} } ,\quad - 1 \le R^{2} \le 1.$$

### Comparing with other functions

To compare the proposed model's performance, the function type is replaced with the sine type function for constraint (6). Changing the constraint is still the model becomes Linear Programming (LP). As a result, this type of function is generated to control the performance of the main model:

$${\text{min}}\,\,RRMAD = \beta \sum\limits_{i} {w_{i} \overline{\Gamma }_{i} } + (1 - \beta )\sqrt { - 2\ln (\alpha )} \sum\limits_{i} {w_{i} \overline{\sigma }_{i} } ,$$

subject to

20 $$f_{s} (x_{i} ) = \sum\limits_{k} {a_{k} \sin (\omega x_{i} )^{k - 1} } \quad \, \begin{array}{*{20}c} {\text{Type 2}} \\ {\text{Sine type function}} \\ \end{array} { 1}\quad \forall i,s,$$

Or

21 $$f_{s} (x_{i} ) = \sum\limits_{k} {a_{k} (x_{i} \sin (\omega x_{i} ))^{k - 1} } \quad \begin{array}{*{20}c} {\text{Type 3}} \\ {\text{Sine type function 2}} \\ \end{array} \, \quad \forall i,s,$$

Or

22 $$f_{s} (x_{i} ) = \sum\limits_{k} {a_{k} (x_{i} + \sin (\omega x_{i} ))^{k - 1} } \quad \begin{array}{*{20}c} {\text{Type 4}} \\ {\text{Sine type function 3}} \\ \end{array} \, \quad \forall i,s,$$

Constraints (3), (5)–(13).

## Results and discussion

**Figure 4:**
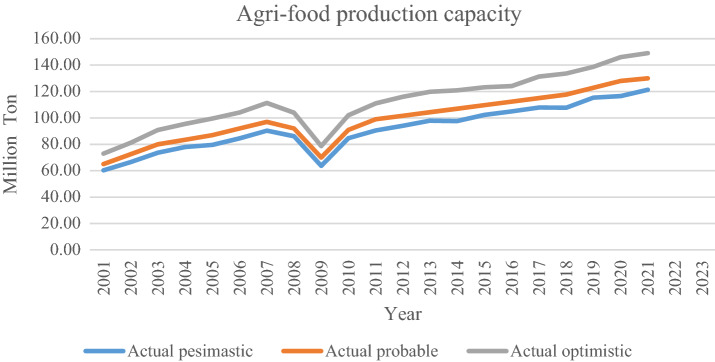
Agri-food production.

**Figure 5:**
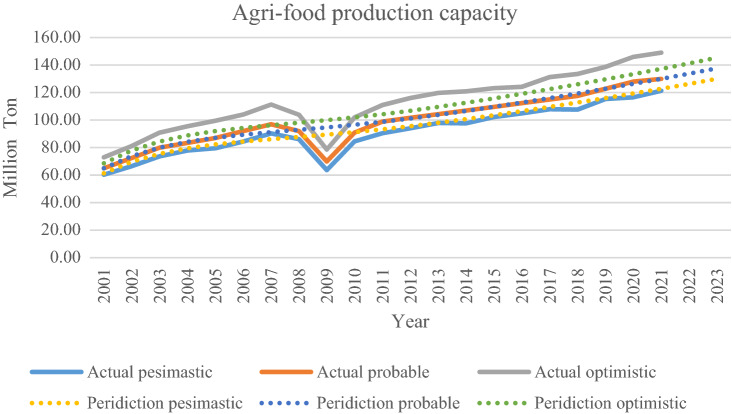
Agri-food production with polynomial regression.

**Figure 6:**
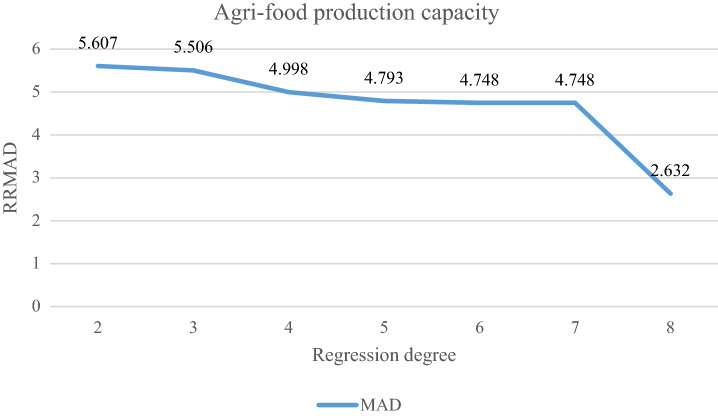
Amount of RRMAD for Agri-food production.

This research's case study concerns agri-food in Iran. Through conversations with agricultural managers, the value of the parameters was determined and is presented in Table 2. The configuration that is applied for solving models with GAMS (CPLEX solver) is as follows: Intel(R), Core(TM) i5-4210U, CPU @ 1.70 GHz, 2.40 GHz, 6.00 GB RAM, and a 64-bit operating system. The number of sets is determined in Table 3. The probability of scenario includes pessimistic, possible, and optimistic scenarios with the same value. So, the volume of agri-food production with uncertainty is shown in Iran (cf. Fig. 4). After obtaining the optimal solution for the model, The RRMAD function is 4.698 in Table 3, and the final function coefficients are determined in Table 4 and Fig. 5. Finally, it is obtained optimal polynomial regression with degree seven in Fig. 6.

**Table 2:** Determining the value of parameters.

Parameters	Value	Unit	Parameters	Value	Unit
$x_{i}$	[2001–2021]	Year	$\beta$	95	%
$y_{is}$	Based on chart	Million Ton	$\alpha$	5	%
$p_{s}$	1/$\left| S \right|$	%	$\lambda$	50	%
$\rho_{s}$	85 + 5.($s$ − 1)	%	$\omega$	$\frac{2\pi }{{12}}$	–

*U* uniform function

**Table 3:** Sets, variables, and constraints, for the case study.

Problem	$\left| I \right|.\left| K \right|.\left| S \right|$	Variable			Constraint	*RRMAD*	Time solution (s)
		Binary	Positive	Free			
P1	21.7.3	0	252	239	358	4.748	0.135

**Table 4:** Amount of coefficient, RRMAD, and *R-squared* for agri-food production.

$\left| K \right|$	$a_{1}$	$a_{2}$	$a_{3}$	$a_{4}$	$a_{5}$	$a_{6}$	$a_{7}$	$a_{8}$	*RRMAD*	*R*^*2*^	Feasibility
2	78.22	3.07							5.607	0.829	Yes
3	82.09	2.09	0.04						5.506	0.831	Yes
4	69.78	7.56	−0.52	0.02					4.998	0.838	Yes
5	60.99	12.64	−1.49	0.09	−2.0E−03				4.793	0.852	Yes
6	58.37	16.25	−2.62	0.22	−9.0E−03	1.3E−04			4.748	0.856	Yes
**7**	**58.19**	**16.56**	−**2.78**	**0.26**	−**1.2E**−**02**	**2.8E**−**04**	−**2.7E**−**06**		**4.748**	**0.855**	**Yes**
8	66.69	2.84	3.47	−0.86	7.6E−02	−3.0E−03	2.8E−05	2.8E−07	2.632	0.911	No

Significant values are in bold.

### Comparing models

**Figure 7:**
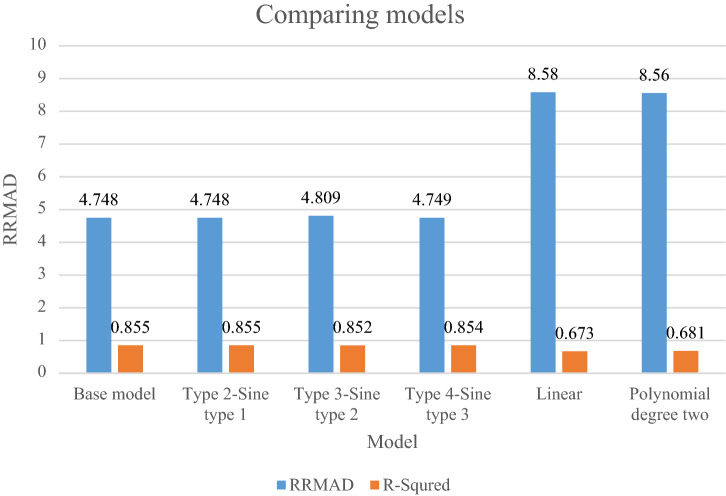
Comparing models.

In this section, the main model is compared with other sine types that are defined in section "Comparing with other functions". The amount of RRMAD and *R-squared* is determined in Table 5 and Fig. 7. As shown, the value of RRMAD of the main model is 1.28% less than other sine types. This mathematical model is better than linear and polynomial degree two regression in RRMAD and *R-squared*.

**Table 5:** Comparing main models with other models.

Reference	Model	$\left| K \right|$	$\omega$	*RRMAD*	Variation	*R*^*2*^
**This research**	**Base model**	**7**	**–**	**4.748**	**–**	**0.855**
	Type 2-Sine type 1	7	$\frac{2\pi }{1}$	4.748	0.00%	0.855
	Type 3-Sine type 2	7	$\frac{2\pi }{{0.5}}$	4.809	1.28%	0.852
	Type 4-Sine type 3	7	$\frac{2\pi }{2}$	4.749	0.02%	0.854
^31^	Linear $\beta = 100\% ,\lambda = 50\%$	2	–	8.58	80.7%	0.673
^32^	Polynomial degree two $\beta = 100\% ,\lambda = 50\%$	3	–	8.56	80.2%	0.681

Significant values are in bold.

### Analyzing the conservativity coefficient

**Figure 8:**
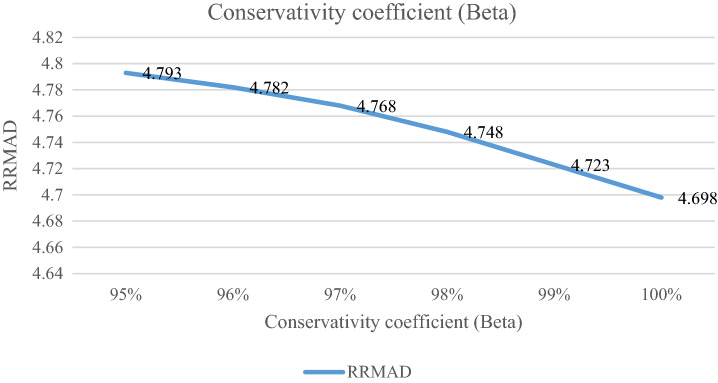
RRMAD for conservativity coefficient.

**Figure 9:**
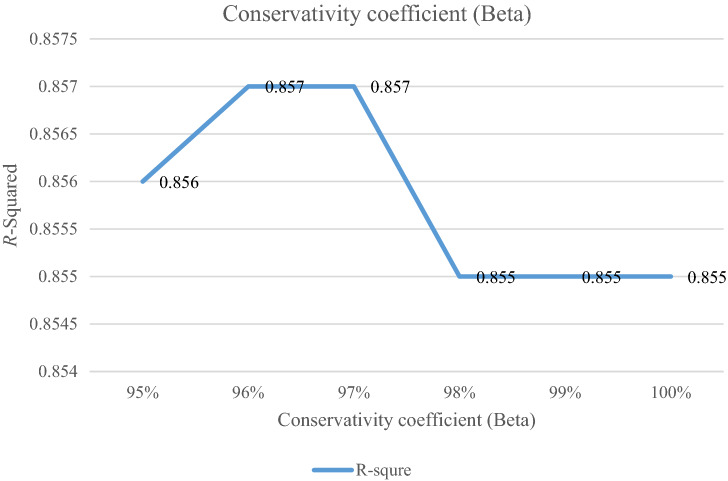
*R-squared* for conservativity coefficient.

The conservativity coefficient ($\beta$) is the preference of decision-makers. It is varied in the range of 95%-100%. When this factor increases to 100%, the RRMAD function decreases in Table 6 and Fig. 8 and if this factor increases by 5%, the RRMAD function will change by about −1.05%, and *R-squared* will fluctuate in Fig. 9, too.

**Table 6:** Analyzing of conservativity coefficient.

Problem	Conservativity coefficient ($\beta$)	*RRMAD*	Variation	*R*^*2*^
P1	95%	4.793	0.95%	0.856
P1	96%	4.782	0.72%	0.857
P1	97%	4.768	0.42%	0.857
**P1-main model**	**98%**	**4.748**	**0.00%**	**0.855**
P1	99%	4.723	-0.53%	0.855
P1	100%	4.698	-1.05%	0.855

Significant values are in bold.

### Analyzing the confidence level

**Figure 10:**
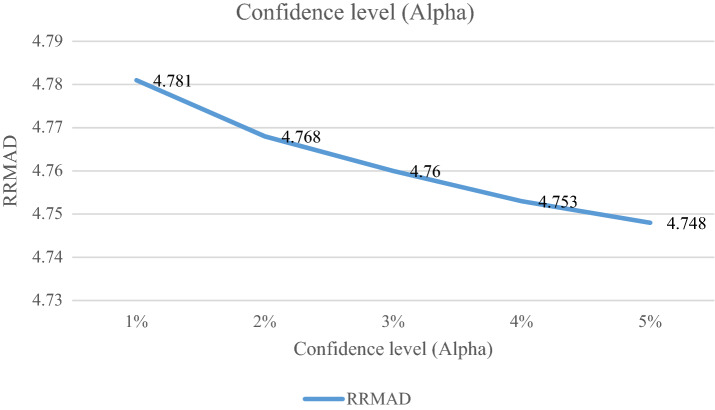
Analyzing the confidence level.

The confidence level of decision-maker is denoted by confidence level ($\alpha$). It is varied between 1 and 5%. If it decreases, the cost function will change to up (cf. Table 7 and Fig. 10). By reducing it to 1%, the cost function grows 0.70%, and *R-squared* is not changed significantly.

**Table 7:** Analyzing the confidence level.

Problem	Confidence level	*RRMAD*	Variation	*R*^*2*^
P1	1%	4.781	0.70%	0.857
P1	2%	4.768	0.42%	0.855
P1	3%	4.760	0.25%	0.855
P1	4%	4.753	0.11%	0.855
**P1-main model**	**5%**	**4.748**	**0.00%**	**0.855**

Significant values are in bold.

### Analyzing weight factor for old and new data

**Figure 11:**
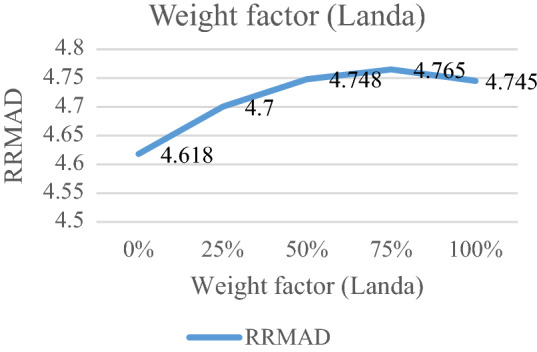
RRMAD for weight factor.

**Figure 12:**
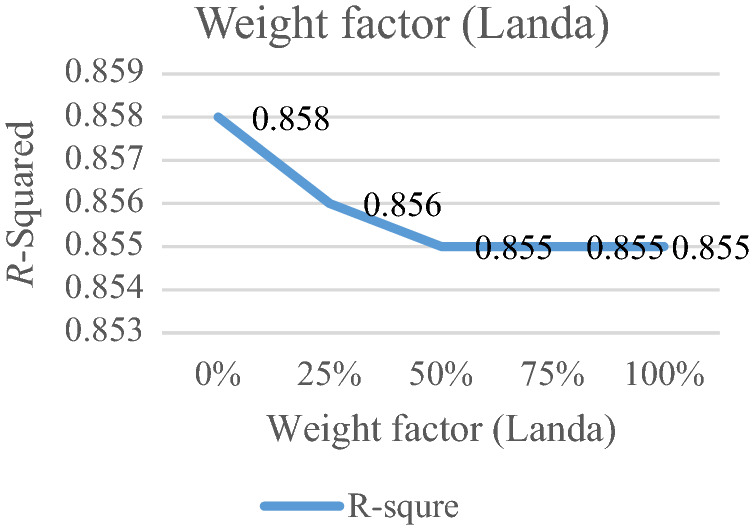
*R-squared* for weight factor.

The weight factor ($\lambda$) is a significant factor for each data. This weight factor is changed between 0 and 100%. When the significant factor is 0%, the substantial factor for all data are $w_{i} = \frac{\left| I \right| - (i - 1)}{{\left| I \right|(\left| I \right| + 1)/2}}$. It means that old data is more important than new data. When the weight factor is 100%. The significant factor of all data is $w_{i} = \frac{i}{\left| I \right|(\left| I \right| + 1)/2},$ that new data are more important than old data. If this coefficient grows, the RRMAD will increase, and *R-squared* will move down smoothly (cf. Figs. 11, 12, and Table 8).

**Table 8:** Analyzing weight factor.

Problem	Weight factor ($\lambda$)	*RRMAD*	Variation	*R*^*2*^
P1	0%	4.618	−2.74%	0.858
P1	25%	4.7	−1.01%	0.856
**P1-main model**	**50%**	**4.748**	**0.00%**	**0.855**
P1	75%	4.765	0.36%	0.855
P1	100%	4.745	-0.06%	0.855

Significant values are in bold.

### Analyzing the resiliency coefficient

**Figure 13:**
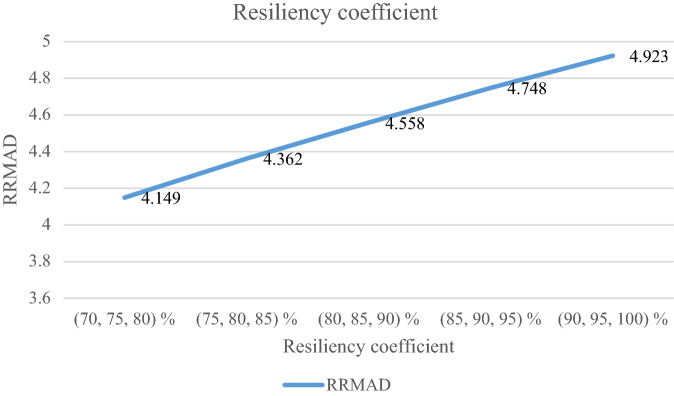
RRMAD for resiliency coefficient.

**Figure 14:**
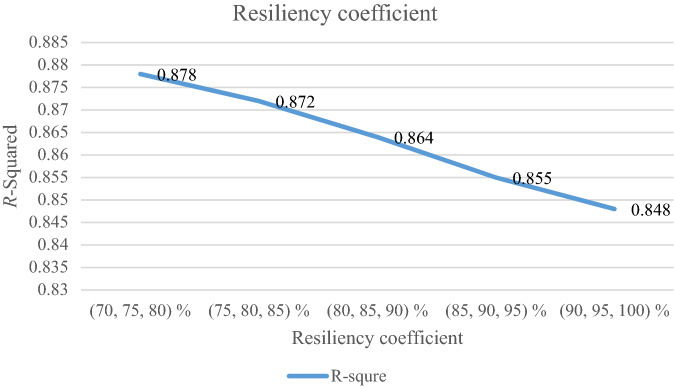
R-squared for resiliency coefficient.

The resiliency coefficient ($\rho_{s}$) as a significant factor for the resiliency situation in the proposed model is analyzed. The RRMAD function increases and the *R-squared* decreases by increasing the resiliency coefficient (cf. Table 9). When the resiliency coefficient increases by 5%, the RRMAD function rises by 3.69% (cf. Figs. 13 and 14).

**Table 9:** Analyzing the resiliency coefficient.

Problem	Resiliency coefficient ($\rho_{s}$)	*RRMAD*	Variation	*R*^*2*^
P1	(70, 75, 80) %	4.149	−12.62%	0.878
P1	(75, 80, 85) %	4.362	−8.13%	0.872
P1	(80, 85, 90) %	4.558	−4.00%	0.864
**P1-main model**	**(85, 90, 95) %**	**4.748**	**0.00%**	**0.855**
P1	(90, 95, 100) %	4.923	3.69%	0.848

Significant values are in bold.

### Analyzing the probability of scenario

**Figure 15:**
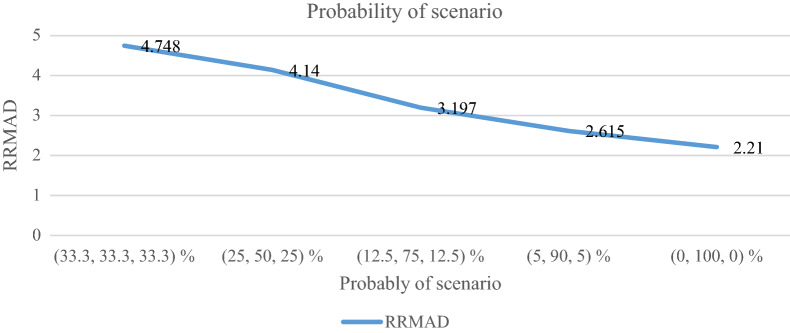
RRMAD for the probability of scenario.

**Figure 16:**
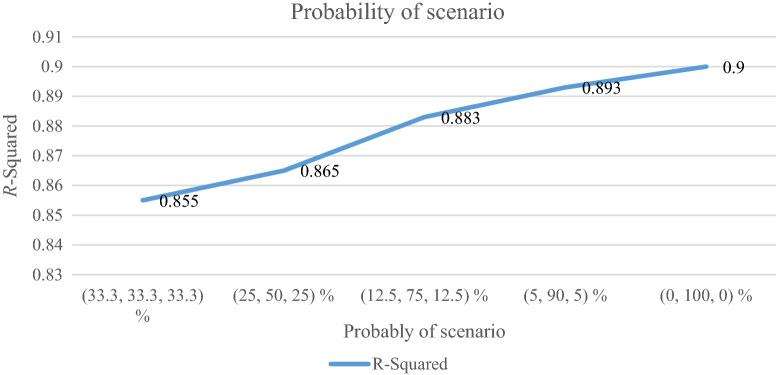
R-squared for the probability of scenario.

The probability of scenario ($p_{s}$) as the probability occurring is analyzed in the regression model. The RRMAD function moves down, and *R-squared* increases by changing the scenario possibility (cf. Table 10). When the scenario possibility increases by 67%, the RRMAD function moves down by 53%, and *R-squared* moves up by 5.6% (cf. Figs. 15 and 16).

**Table 10:** Analyzing the probability of scenario.

Problem	Probability of scenario ($p_{s}$)	*RRMAD*	Variation *RRMAD*	*R*^*2*^	Variation *R*^*2*^
**P1-main model**	**(33.3, 33.3, 33.3) %**	**4.748**	**0.00%**	**0.855**	**0.00%**
P1	(25, 50, 25) %	4.140	−12.81%	0.865	1.17%
P1	(12.5, 75, 12.5) %	3.197	−32.67%	0.883	3.27%
P1	(5, 90, 5) %	2.615	−44.92%	0.893	4.44%
P1	(0, 100, 0) %	2.210	−53.45%	0.900	5.26%

Significant values are in bold.

## Discussion

This study examined a RRML for forecasting agri-food production and is the first to combine the concepts of robustness and resiliency for this problem. To deal with uncertainty, this study employed a scenario-based approach. Furthermore, this problem considers disruption-based flexibility as resiliency in ML for forecasting and compares the proposed model to other functions to demonstrate the model's performance.

After solving the model, the model obtains the coefficient of the proposed function in the RRML approach. The proposed model is compared with other sine-type functions and found that the model's performance is better than types of sine functions, and RRMAD is less than them. Eventually, by embedding robustness and resiliency concepts, this research considers uncertainty that did not pay attention to previous research in the ML model. This research tries to develop previous work^22^ robustness and resiliency concepts with a scenario-based approach. Resiliency concepts were not considered in previous work, but this research considers this concept to survey uncertainty disruption in the ML model. In addition, the main model is compared with sine types that are defined in section "Comparing with other functions". The amount of RRMAD and *R-squared* is determined in Table 5 and Fig. 7. As can be seen, the value of RRMAD of the main model is 1.28% less than other sine-type. The conservativity coefficient is varied in the range of 95–100%. When this factor increases to 100%, the RRMAD function decreases in Table 6, Fig. 8. When this factor increases by 5%, the RRMAD function will change by about −1.05%, and *R-squared* will fluctuate in Fig. 9, too. The confidence level is varied between 1 and 5%. If it decreases, the cost function will change to up (cf. Table 7 and Fig. 10). By reducing it to 1%, the cost function grows 0.70%. As can be seen, *R-squared* is not changed significantly. The significant factor is changed from 0 to 100%. When the significant factor is 0%, old data is more important than new data. When the significant factor is 100%, new data is more important than old data. If this coefficient grows, the RRMAD will increase, and *R*-squared will move down smoothly (cf. Figs. 11, 12, and Table 8). The resiliency coefficient is analyzed as a significant factor for the resiliency situation in the regression model. The RRMAD function increases and *R-squared* decreases by increasing the resiliency coefficient (cf. Table 9). When the resiliency coefficient increases by 5%, the RRMAD function rises by 3.69% (cf. Figs. 13 and 14). The scenario probability is analyzed as the probability of occurring in the regression model. The RRMAD function moves down, and *R*-squared increases by changing the scenario's probability (cf. Table 10). When the scenario probability increases by 67%, the RRMAD function moves down by 53%, and *R-squared* rise by 5.6% (cf. Figs. 15 and 16).

Therefore, sensitivity analyses are run for essential parameters. As a result, it is suitable to embed robustness and resiliency concepts for this problem because these concepts improve the model's performance and make the model robust and resilient against disruption.

## Managerial insights and practical implications

This research pays attention to predicting agri-food capacity production. Therefore, a novel ML approach is utilized for the first time. The robustness and resiliency concepts are combined in this approach. Robust scenario-based optimization is used to cope with an uncertain situation. This method applies flexibility based on disruption as a resiliency strategy. In addition, the proposed model is compared with other models to show the model's performance. The model's performance is suitable for forecasting agri-food capacity production. Eventually, it is suggested to managers and decision-makers of agri-food to use this style of mathematical model to predict volume production. This model helps the decision-maker to have better decisions.

## Conclusions and outlook

This research proposes a new framework for agri-food capacity production by considering resiliency and robustness and paying attention to disruption and risk for the first time. A robust stochastic optimization is applied by adding robustness to the objective function and resiliency situation in constraint. This model minimizes a predicted linear function's MAD and standard deviation coefficient in agri-food production. This model is suggested to managers and decision-makers of agri-food to apply for forecasting production. This model help to improve the performance of decision maker.

Therefore, the results are as follows:

1. The main model is compared with other sine-type functions defined in section "Comparing with other functions". The amount of RRMAD and *R-squared* is determined in Table 5 and Fig. 7. As can be seen, the value of RRMAD is 1.28% less than other sine types.
2. This research varied conservativity coefficient in the range of 95–100%. When this factor increases to 100%, the RRMAD function decreases in Table 6, Fig. 8. When this factor increases by 5%, the RRMAD function will change by about −1.05%, and *R-squared* will fluctuate in Fig. 9, too.
3. In addition, confidence levels are varied between 1 and 5%. If it decreases, the cost function will change to up (cf. Table 7 and Fig. 10). By reducing it to 1%, the cost function grows 0.70%. As can be seen, *R-squared* is not changed significantly.
4. The weight factor is changed from 0 to 100%. When the significant factor is 0%, old data is more important than new data. When the weight factor is 100%, new data is more important than old data. If this coefficient grows, the RRMAD will increase, and *R*-squared will move down smoothly (cf. Figs. 11, 12, and Table 8).
5. The resiliency coefficient is analyzed as a significant factor for the resiliency situation in the regression model. The RRMAD function increases, and *R-squared* decrease by increasing the resiliency coefficient (cf. Table 9). When the resiliency coefficient increases by 5%, the RRMAD function rises by 3.69% (cf. Figs. 15 and 16).
6. The scenario probability is analyzed as a scenario occurring in the regression model. The RRMAD function moves down, and *R-squared* increases by changing the scenario probability (cf. Table 10). When the possibility of the scenario increases by 67%, the RRMAD function moves down by 53% and *R-squared* up by 5.6% (cf. Figs. 13 and 14).

This model is completely LP and lacks any binary variables. As a result, this problem can be solved on a large scale in polynomial time. Consequently, increasing the scenario induces the time to grow polynomially. There is, therefore, no substantial restriction on a large scale.

Eventually, it is suggested to use other antifragility or resiliency strategies. These strategies include additional functions for learning as antifragility strategies in the ML model. Further, it is proposed to use risk criteria like Conditional and Entropic Value at Risk (CVaR or EVaR) to add risk fluctuation^33,34^. Other uncertain approaches like stochastic programming and robust optimization (convex) make it close to the real world^35^. In addition, the fuzzy and DDRO methods have been helpful for risk-averse decision-makers in the recent decade.

## Data Availability

The datasets generated and/or analysed during the current study are available in the RRMLFAFP^36^ repository, http://dx.doi.org/10.17632/z53s5dtgpb.1.

## References

[1] Penalba M, Aizpurua JI, Martinez-Perurena A, Iglesias G (2022). A data-driven long-term metocean data forecasting approach for the design of marine renewable energy systems. Renew. Sustain. Energy Rev..

[2] Somu N, Ramamritham K (2021). A deep learning framework for building energy consumption forecast. Renew. Sustain. Energy Rev..

[3] Rezapour M, Hansen L (2022). A machine learning analysis of COVID-19 mental health data. Sci. Rep..

[4] Kang H, An J, Kim H, Ji C, Hong T, Lee S (2021). Changes in energy consumption according to building use type under COVID-19 pandemic in South Korea. Renew. Sustain. Energy Rev..

[5] Mourtzinis S, Esker PD, Specht JE, Conley SP (2021). Advancing agricultural research using machine learning algorithms. Sci. Rep..

[6] Yoon H-J, Seo S-K, Lee C-J (2022). Multi-period optimization of hydrogen supply chain utilizing natural gas pipelines and byproduct hydrogen. Renew. Sustain. Energy Rev..

[7] Salehi-Amiri A, Zahedi A, Akbapour N, Hajiaghaei-Keshteli M (2021). Designing a sustainable closed-loop supply chain network for walnut industry. Renew. Sustain. Energy Rev..

[8] Nili M, Seyedhosseini SM, Jabalameli MS, Dehghani E (2021). A multi-objective optimization model to sustainable closed-loop solar photovoltaic supply chain network design: A case study in Iran. Renew. Sustain. Energy Rev..

[9] Tsakanikas P, Karnavas A, Panagou EZ, Nychas G-J (2020). A machine learning workflow for raw food spectroscopic classification in a future industry. Sci. Rep..

[10] Jeff, L. *Fertile Ground: Ontario’s Agri-Food Industry Delivers Sustainable Growth*.

[11] Tawn R, Browell J (2022). A review of very short-term wind and solar power forecasting. Renew. Sustain. Energy Rev..

[12] Huang B, Liu J, Jiao J, Lu J, Lv D, Mao J (2022). Applications of machine learning in pine nuts classification. Sci. Rep..

[13] Uddin S, Ong S, Lu H (2022). Machine learning in project analytics: A data-driven framework and case study. Sci. Rep..

[14] Kantasa-Ard A, Nouiri M, Bekrar A, Aitel Cadi A, Sallez Y (2021). Machine learning for demand forecasting in the physical internet: a case study of agricultural products in Thailand. Int. J. Prod. Res..

[15] Pereira LN, Cerqueira V (2021). Forecasting hotel demand for revenue management using machine learning regression methods. Curr. Issues Tour..

[16] Kohli S, Godwin GT, Urolagin S (2021). Sales prediction using linear and KNN regression. Adv. Mach. Learn. Comput. Intell. (Springer).

[17] Samar Ali S, Kaur R, Ersöz F, Lotero L, Weber G-W (2019). Evaluation of the effectiveness of green practices in manufacturing sector using CHAID analysis. J. Remanuf..

[18] Ali SS, Kaur R, Persis DJ, Saha R, Pattusamy M, Sreedharan VR (2020). Developing a hybrid evaluation approach for the low carbon performance on sustainable manufacturing environment. Ann. Oper. Res..

[19] Papacharalampous G, Langousis A (2022). Probabilistic water demand forecasting using quantile regression algorithms. Water Resour. Res..

[20] Balaji Prabhu, B.V., & Dakshayini, M. *Computational Performance Analysis of Neural Network and Regression Models in Forecasting the Societal Demand for Agricultural Food Harvests*. 1287–300 (Research Anthology on Artificial Neural Network Applications: IGI Global, 2022).

[21] Baryannis G, Dani S, Antoniou G (2019). Predicting supply chain risks using machine learning: The trade-off between performance and interpretability. Futur. Gener. Comput. Syst..

[22] Lotfi R, Kheiri K, Sadeghi A, BabaeeTirkolaee E (2020). An extended robust mathematical model to project the course of COVID-19 epidemic in Iran. Ann. Oper. Res..

[23] Carbonneau R, Laframboise K, Vahidov R (2008). Application of machine learning techniques for supply chain demand forecasting. Eur. J. Oper. Res..

[24] Fradinata E, Kesuma ZM, Rusdiana S (2018). Support vector regression and adaptive neuro fuzzy to measure the Bullwhip effect in supply chain. J. Phys. Conf. Ser. (IOP Publishing).

[25] Al-Musaylh MS, Deo RC, Li Y, Adamowski JF (2018). Two-phase particle swarm optimized-support vector regression hybrid model integrated with improved empirical mode decomposition with adaptive noise for multiple-horizon electricity demand forecasting. Appl. Energy.

[26] Priyadarshi R, Panigrahi A, Routroy S, Garg GK (2019). Demand forecasting at retail stage for selected vegetables: A performance analysis. J. Model. Manag..

[27] Kilimci ZH, Akyuz AO, Uysal M, Akyokus S, Uysal MO, Atak Bulbul B (2019). An improved demand forecasting model using deep learning approach and proposed decision integration strategy for supply chain. Complexity.

[28] Phyo PP, Jeenanunta C (2021). Daily load forecasting based on a combination of classification and regression tree and deep belief network. IEEE Access..

[29] Yucesan M, Pekel E, Celik E, Gul M, Serin F (2021). Forecasting daily natural gas consumption with regression, time series and machine learning based methods. Energy Sources Part A Recov. Utiliz. Environ. Effects.

[30] Feizabadi J (2022). Machine learning demand forecasting and supply chain performance. Int. J. Log. Res. Appl..

[31] Glover F (1975). Improved linear integer programming formulations of nonlinear integer problems. Manag. Sci..

[32] Freedman DA (2009). Statistical Models: Theory and Practice.

[33] Zare Mehrjerdi Y, Lotfi R (2019). Development of a mathematical model for sustainable closed-loop supply chain with efficiency and resilience systematic framework. Int. J. Supply Oper. Manag..

[34] Lotfi R, Kargar B, Hoseini SH, Nazari S, Safavi S, Weber GW (2021). Resilience and sustainable supply chain network design by considering renewable energy. Int. J. Energy Res..

[35] Lotfi R, Mardani N, Weber GW (2021). Robust bi-level programming for renewable energy location. Int. J. Energy Res..

[36] Lotfi R. *RRMLFAFP 2022. *10.17632/z53s5dtgpb.1 (2022).

